# Epistemic spillovers: Learning others’ political views reduces the ability to assess and use their expertise in nonpolitical domains

**DOI:** 10.1016/j.cognition.2018.10.003

**Published:** 2019-07

**Authors:** Joseph Marks, Eloise Copland, Eleanor Loh, Cass R. Sunstein, Tali Sharot

**Affiliations:** a Affective Brain Lab, Experimental Psychology, University College London, London, UK; b Harvard Law School, Harvard University, Cambridge, MA, USA

**Keywords:** Information-seeking, Political homophily, Influence

## Abstract

On political questions, many people prefer to consult and learn from those whose political views are similar to their own, thus creating a risk of echo chambers or information cocoons. We test whether the tendency to prefer knowledge from the politically like-minded generalizes to domains that have nothing to do with politics, even when evidence indicates that politically like-minded people are less skilled in those domains than people with dissimilar political views. Participants had multiple opportunities to learn about others’ (1) political opinions and (2) ability to categorize geometric shapes. They then decided to whom to turn for advice when solving an incentivized shape categorization task. We find that participants falsely concluded that politically like-minded others were better at categorizing shapes and thus chose to hear from them. Participants were also more influenced by politically like-minded others, even when they had good reason not to be. These results replicate in two independent samples. The findings demonstrate that knowing about others’ political views interferes with the ability to learn about their competency in unrelated tasks, leading to suboptimal information-seeking decisions and errors in judgement. Our findings have implications for political polarization and social learning in the midst of political divisions.

## Highlights

• Participants believe politically like-minded others are better at unrelated tasks.
• They turn to the politically like-minded even when others are more accurate.
• Participants are more influenced by politically like-minded others on those issues.

## 1 Introduction

To make good choices, human beings turn to one another for information (Gino et al., 2012, Hofmann et al., 2009, Schrah et al., 2006, Yaniv and Kleinberger, 2000). When selecting a retirement plan or deciding whether to grab an umbrella on the way out, people are motivated to get information from the most accurate source. Obviously, people would prefer to receive a weather report from the weather forecaster whose predictions are 80% correct than from the one who is wrong every other day.

At the same time, people also prefer to receive information from others who are similar to themselves. Democrats are more likely to turn to CNN for their news and Republicans to Fox News for their daily updates (The Pew Research Center, 2009). This is partly because people assume that like-minded people are more likely to be correct – a phenomenon that can lead to echo chambers (Del Vicario et al., 2016, Sunstein, 2017). But if people had clear and repeated opportunities to learn who is right and who is wrong, would similarity interfere with the ability to learn about accuracy?

It has been suggested that people assess others’ expertise based on their own beliefs (Boorman et al., 2013, Faraji-Rad et al., 2012, Faraji-Rad et al., 2015, Schilbach et al., 2013). In one study (Boorman et al., 2013) participants were asked to evaluate financial assets while also observing the judgments made by others before receiving feedback. The findings indicated that participants updated their beliefs about others’ expertise not only after receiving feedback about the asset’s value, but also *before* feedback was available. In particular, participants took into account their own judgment about the asset when updating their assessment of the other participant’s ability on the task. When the other person’s judgment was in accord with their own, they gave the other person credit, but they penalized that person when their judgments conflicted. In fact, subjects gave considerable credit to people for correct judgements with which they *agreed*, but barely gave them any credit at all for accurate judgments with which they *disagreed*. This bias interferes with the ability to assess others’ skills, leading individuals to conclude that people who think like them about a certain topic are more likely to be experts.

Our question, however, is whether similarity in one field will generalize to a biased assessment in another field – a kind of epistemic spillover. If we conclude that person X is good at finance simply because he tends to agree with us about the value of stocks, will we then be more likely to conclude he has superior abilities in predicting the weather? Because of the halo effect (Dion et al., 1972, Nisbett and Wilson, 1977, Thorndike, 1920), which is the tendency for an evaluation in one area to influence an evaluation in another area, we predicted this to be the case. The likely downstream behavioural consequence is that people will turn to others who think like them in one area for information in another area, even in cases where the evidence in front of them clearly indicates that this is suboptimal.

Here, we ask whether (dis)similarity in political views interferes with the ability to learn about another person’s competency in an unrelated task (specifically categorizing shapes) in a situation in which it is in people’s best interest to learn who excels in the task in order to turn to them for assistance. In the first part of our experiment, participants had an opportunity to learn whether others (i) had similar political opinions to theirs and (ii) how well they did in a task that required learning about shapes. After rating others on these two characteristics, they completed the second part of the experiment, where they decided to whom to turn to for advice when solving the shape task. They were rewarded for accuracy on the task and thus had an economic incentive to turn to the participant who was most skilled at the task.

We find that (dis)similarity in political views interferes with the ability to make an accurate assessment of people’s expertise in the domain of shapes, which leads to two central outcomes. The first is that people chose to hear about shapes from others who are politically like-minded, even when those people are not especially good at the shape task, rather than to hear from people who excel at the shape task but have different political opinions. The second is that people are more influenced by those with similar political opinions, even when they had the opportunity to learn that those by whom they are influenced are not especially good at the task they are solving. The results replicate in two independent samples. We suspect that these findings can be found in the real world, and that they help explain a range of phenomena, including the spread of fake news (Friggeri et al., 2014, Kahne and Bowyer, 2017), conspiracy theories (Del Vicario et al., 2016), polarization (Druckman et al., 2013, Prior, 2007), and insufficient learning in general (Yaniv and Kleinberger, 2000, Yaniv and Milyavsky, 2007).

## 2 Experiment 1

### 2.1 Method

#### 2.1.1 Participants

American residents over 18 years of age who speak English were recruited on Amazon Mechanical Turk. All participants provided demographic information (see supplementary materials). Sample size was determined using a power analysis based on a pilot study.

154 participants completed the first part of the task (Learning Stage). Participants had to pass the learning stage test (see below) in order to continue to the choice stage. 97 participants (34 females and 63 males, aged 20–58 years *M* = 34.81, *SD* = 9.59) passed the learning test. Participants who passed the learning test did not differ from those who failed on age, gender, ethnicity, language, education, income, subjective socio-economic-position, political ideology, interest/involvement in US politics, or generalized trust (all P > .12).

All participants were paid a base rate of $2.50. They were told they could earn a bonus between $2.50 and $7.50 based on their performance, but were not told exactly how performance would be measured. Unbeknownst to the participants our rule for paying the bonus was as follows: any participants that passed the learning stage test (see details below) and completed the choice stage received $5 bonus.

#### 2.1.2 Study design

##### 2.1.2.1 Learning stage

The goal of the learning stage was to give participants an opportunity to learn about the other participants’ (hereafter ‘***sources***’) political views and about their competency on the shape task (hereafter ‘Blap task’). Before the learning stage, participants completed four practice blap trials and four practice political trials. They were not presented with information from sources on practice trials.

The learning stage consisted of 8 blocks of 20 trials each (10 blap trials and 10 political trials interleaved). Responses from one of the four sources were shown for the duration of a block (each source was used in two blocks), the order of which was randomized across blocks. Qualtrics’ *loop and merge* tool was used to randomize the order of the questions within each block.

1. ***Blap trials* (**Fig. 1a). On each trial, one of 204 coloured shapes was presented on screen. Participants were required to learn through trial and error to classify shapes as ‘blaps’ or ‘not blaps’, ostensibly based on the shape’s features. Unbeknownst to the participants, whether a shape was a blap or not was not rule based, but rather randomly determined before the beginning of the task, such that half the stimuli were categorized as “blaps”. Because participants did not in fact have any means to learn which type of stimulus was a blap, the average performance across participants was around 50% (*M* = 48%, *SD* = 10.57). Participants had as much time as they needed to enter their response with a key press indicating either “yes” (the shape is a blap) or “no” (it is not) (*M* = 2.78 s, *SD* = 9.27). They then observed for 1sc the response of one of the four sources. Thereafter they received feedback on whether they and the source were each correct or incorrect (2sc).

**Fig. 1:**
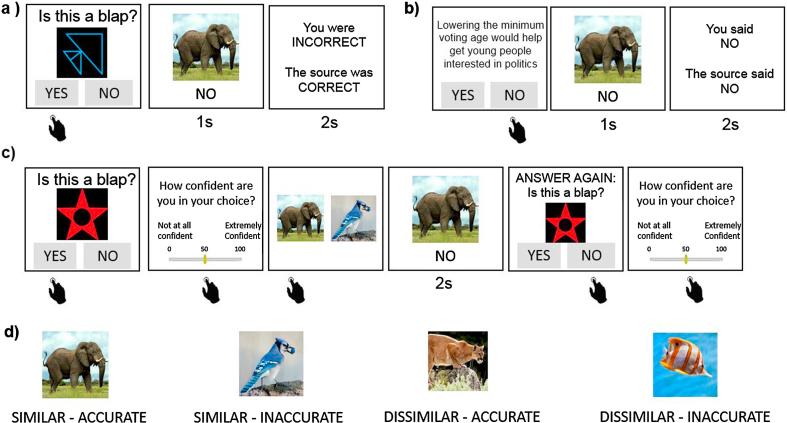
Task. During the Learning Stage participants learned about the political opinions of four sources (represented by an animal photo) and about the sources’ accuracy on a shape task (blap task). (a) Blap trials and (b) political trials were interleaved. (a) On each blap trial a novel shape was presented and the participants had to indicate whether they believed the shape was a blap (yes or no). They then saw the response of one of four sources represented by an animal photo. This was followed by feedback. (b) On political trials a political statement was presented and the participants had to indicate whether they agreed with it (yes or no). They then saw the response of one of four sources represented by an animal photo. This was followed by a reminder of their response and the source’s response. (c) During the Choice Stage participants completed blap trials only. On each blap trial a novel shape was presented and the participant had to indicate whether they believed the shape was a blap (yes or no) and enter a confidence rating. They were then presented with two sources and asked to choose whose answer they would like to see. They then saw the response of the chosen source. Finally, they were given a chance to update their initial answer and confidence rating. Responses were self-paced unless otherwise stated. (d) There were four sources represented with animal photos which the participants were led to believe were other participants but were in fact algorithms designed to respond in the following pattern: (i) One source agreed with the subject on 80% of the political trials and was correct on 80% of blap trials (***Similar-Accurate***), (ii) One source agreed with the subject on 80% of the political trials and was correct on only 50% of blap trials (*Similar-Random*), (iii) One source agreed with the subject on 20% of the political trials and was correct on 80% of blap trials (***Dissimilar-Accurate***), and (iv) One source agreed with the subject on 20% of the political trials and was correct on 50% of blap trials (***Dissimilar-Random***). Pictures assigned to sources were *counter-balanced*.
2. ***Political trials*** (Fig. 1b). On political trials, participants indicated whether they agreed or disagreed with one of 84 social/political cause-and-effect statements (e.g. “Lowering the minimum voting age would help get young people interested in politics”, see full set of statements in supplementary materials). These statements were developed on the basis of various political attitude questionnaires (see supplementary materials). Participants had as much time as they needed to press a key button indicating whether their response was “yes” or “no” (*M* = 5.89 s, *SD* = 16.87). They then observed for 1sc the response of one of the four sources. Thereafter they were shown their response together with that of the source (2sc).

##### 2.1.2.2 Sources

Participants were told that on each trial, they would be presented with the response of one of four participants (‘sources’) who performed the task earlier. Unbeknownst to the participants, these sources were not in fact other people but algorithms designed to respond in the following pattern. (i) One source agreed with the subject on 80% of the political trials and was correct on 80% of blap trials (***Similar-Accurate***). (ii) One source agreed with the subject on 80% of the political trials and was correct on only 50% of blap trials (***Similar-Random***). (iii) One source agreed with the subject on 20% of the political trials and was correct on 80% of blap trials (***Dissimilar-Accurate***). (iv) One source agreed with the subject on 20% of the political trials and was correct on 50% of blap trials (***Dissimilar-Random***). On blap trials all sources agreed with the participant about half the time on average (*M* = 50%, *SD* = 11.52). To avoid gender and racial bias, sources were represented with a picture of an animal. Pictures assigned to sources were counter-balanced.

##### 2.1.2.3 Attention check

At the end of each block, participants were presented with an attention check in which they were asked one of the following questions regarding the last trial: “Did the source AGREE or DISAGREE with your answer?”; “What was your last response?”; “Which source was shown on the last trial?”; “Was the last question a political or blap question?” For the latter two questions, 98.97% and 93.81% of participants were correct, respectively. Data was mistakenly not saved to report accuracy of the former two questions.

##### 2.1.2.4 Learning test

The goal of the study was to assess how similarity affected the ability to assess competence and information-seeking behaviour. We thus tested participants’ perception of who was similar to them to determine if the similarity manipulation was successful. Specifically, after the learning stage, participants were presented with 12 trials. On each trial two sources were presented and the subject had to indicate who was more similar to them (“Who is more similar to you?”). Each possible pair of sources (six combinations) was presented twice for a total of twelve trials. Only participants who responded correctly (as determined according to the similarity manipulation described above) on eleven trials or more were considered to have accurately assessed similarity and continued to the choice stage (=97 participants).

##### 2.1.2.5 Ratings of similarity and accuracy

Participants then rated each source on (1) how competent they were at determining if each object was a blap (“How competent was the source at figuring out if each object was a blap?” from 0 = “Very incompetent” to 100 = “Very competent”) and (2) how similar the source was to them (“How similar do you think this source was to you?” from 0 = “Not at all like me” to 100 = “Exactly like me”). We did not specifically ask about political similarity, as we wanted to avoid artificially focusing subjects’ attention on that question. While participants may have construed the question as referring to political similarity and/or similarity on blap performance and/or similarity to the image of the animal, this would have only added noise to the data. As can be observed in the result section, sources who were objectively politically similar to the subjects were rated significantly higher on this scale, as expected.

##### 2.1.2.6 Choice stage (Fig. 1c)

The goal of the choice stage was to assess who the participant wanted to hear from about blaps and how they used the information they received. On each of 120 trials, participants were presented with a novel shape and asked to indicate with a button press whether they thought the shape was a blap (“yes” or “no”) (RT: *M* = 3.46 s, *SD* = 53.90). They subsequently rated their confidence in this decision (self-paced) on a scale from 0 (not at all confident) to 100 (extremely confident). They were then presented with a pair of sources and asked whose response they wanted to see (self-paced) (RT: *M* = 2.04 s, *SD* = 79.13). They were then shown the response of the chosen source for 2 s. Thereafter the shape was presented again and participants were asked again to indicate with a button press whether they believed the shape was a blap (“yes” or “no”) (RT: *M* = 1.29 s, *SD* = 9.79). Lastly, participants rated their confidence (self-paced) in their final decision.

The participants were instructed at the beginning of the choice stage that they could alter their answer on this second guess if they wanted to. There were 6 blocks of 20 trials each with the six source pairs pseudo-randomized throughout each block. There were no political trials nor feedback in the choice stage.

##### 2.1.2.7 Second attention check

As in the learning stage, participants were presented with an attention check question at the end of each block in which they were asked one of the following questions: “Which source did you NOT select on the last trial?”; “Which source did you select on the last trial?”; “Did the source AGREE or DISAGREE with your answer?” There was an error in recording this data, thus we cannot provide accuracy rates.

##### 2.1.2.8 Post-task ratings and debrief

Finally, participants completed a debriefing questionnaire (see supplementary materials). During this debrief, participants were asked once again (1) how competent each source was at determining if each object was a blap (“How competent was the source at figuring out if each object was a blap?” from 0 = “Very incompetent” to 100 = “Very competent”) and (2) how similar the source was to them (“How similar do you think this source was to you?” from 0 = “Not at all like me” to 100 = “Exactly like me”). Analysis of post-task ratings and questions are presented in supplementary material (see supplementary materials).

### 2.2 Results

#### 2.2.1 Participants prefer to receive information about shapes from politically like-minded sources

**Fig. 2:**
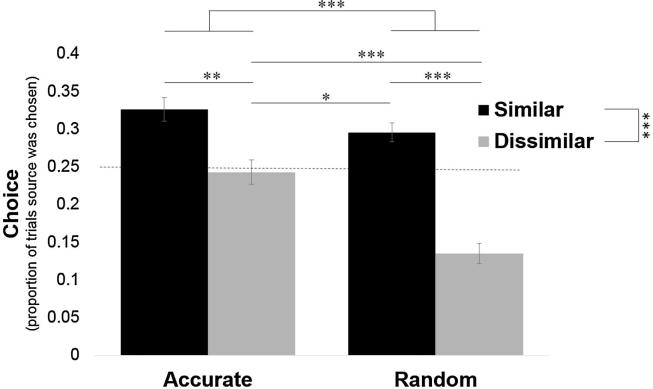
Participants prefer to receive information about shapes from politically like-minded sources. For each participant we calculated the percentage of times they selected to hear from each source about blaps out of all trials and averaged across participants. As each source was presented as an option an equal number of times, if the participants had no preference each source would be selected on about 25% of trials. A preference (main effect) for both accurate sources over inaccurate sources and for politically similar sources over politically dissimilar sources was found. Error bars represent SEM. ^*^p < .05, ^**^p < .01, ^***^p < .001.

We first asked whom participants select to hear from on the blap task. We find that participants sensibly prefer to hear from sources that are more accurate on the blap task, but also prefer to hear from politically like-minded sources even when they were not very good at the blap task (Fig. 2).

Specifically, each source was presented as an option out of two sources on 50% of trials. Thus, if participants had no preference they would select each source on 25% of the trials. We found that the similar-accurate source was chosen most often (*M* = 33%, *SD* = 15.56; significantly greater than chance: t(96) = 4.85, *p* < .001), followed by the similar-random source (*M* = 30%, *SD* = 12.30; significantly greater than chance level: t(96) = 3.65, *p* < .001), followed by the dissimilar-accurate source (*M* = 24%, *SD* = 15.93; not different from chance level: t(96) = −0.44, *p* = .66), and finally by the dissimilar-random source (*M* = 13%, *SD* = 13.53; significantly lower than chance: t(96) = −8.34, *p* < .001). Entering percentage choice into a two (similar/dissimilar) by two (accurate/random) ANOVA revealed a main effect of source accuracy (*F*(1, 96) = 23.32, *p* < .001, η_p_^2^ = 0.20), a main effect of political similarity (*F*(1, 96) = 33.67, *p* < .001, η_p_^2^ = 0.26) and an interaction (*F*(1, 96) = 7.22, *p* = .008, η_p_^2^ = 0.07). The interaction was due to participants selecting to hear from the accurate-dissimilar source over the random-dissimilar source (t(96) = 5.05, *p* < .001, *d* = 0.73), but revealing no preference between the two similar sources (t(96) = 1.62, *p* = .11, *d* = 0.22). Strikingly, participants preferred to hear from the politically like-minded source that performed randomly on the blap task over the source that was accurate on the blap task but dissimilar politically (*t*(96) = −2.10, *p* = .038, *d* = −0.37).

Mean reaction times for source choice were as follows: accurate-similar source (*M* = 3.52 s, *SD* = 138.29); accurate-dissimilar source (*M* = 1.36 s, SD = 5.45); random-similar source (*M* = 1.24 s, *SD* = 4.66), inaccurate-dissimilar source (*M* = 1.44 s, *SD* = 4.24). Entering choice log reaction times into a 2 (similar/dissimilar) by 2 (accurate/random) ANOVA did not reveal any main effects or interaction (all P > .13).

#### 2.2.2 Political similarity leads to an illusory perception of competence on the shape task

What could explain the tendency to seek information about shapes from others who are politically like-minded? Our hypothesis was that (dis)similarity in political views will interfere with participants’ ability to assess others’ competence on the blap task. The rationale is that political (dis)similarity will generate a (negative) positive view of the source, which will generalize to the unrelated domain of shape categorization.

**Fig. 3:**
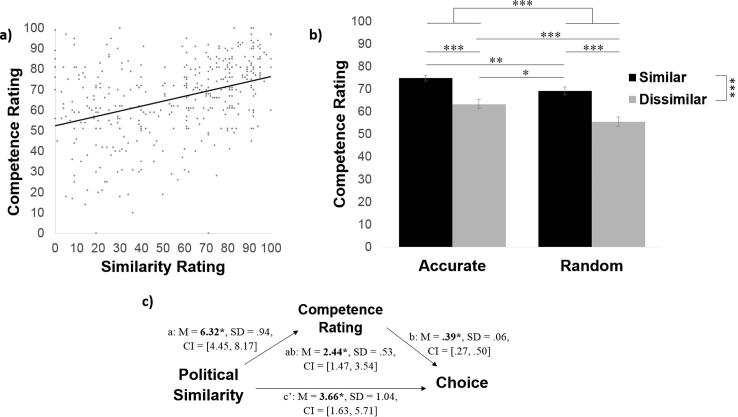
An illusory perception of accuracy mediates the relationship between political similarity and information seeking behavior. (a) The true correlation between how accurate a source was on the blap task and how like-minded they were to the participant was zero. Nevertheless, participants ratings revealed an illusory perception that the two were related (r = 0.37 *p* < .001). (b) Participants rated accurate sources as more competent on the blap task, but also rated politically like-minded sources as more competent on the blap task. (c) A mediation model revealed that perceived competence partially mediated the relationship between political similarity and choice of which source to hear from about blaps. Error bars represent SEM. ^*^p < .05, ^**^p < .01, ^***^p < .001.

To test this hypothesis we first tested for a correlation between participants’ ratings of how similar the sources were to them and how good the sources were on the blap task. The *true* correlation was zero. Nonetheless, participants had an *illusory* perception that the more similar the source was to them, the better the source was on the shape task (r = 0.37, *p* < .001, Fig. 3a).

Second, we examined how participants rated the four sources on their ability to categorize shapes. Entering these ratings into a two (similar/dissimilar) by two (accurate/random) repeated-measures ANOVA revealed not only a sensible main effect of source accuracy (*F*(1, 96) = 22.98, *p* < .001, η_p_^2^ = 0.19), but also an illusory main effect of source political similarity (*F*(1, 96) = 45.41, *p* < .001, η_p_^2^ = 0.32) and no interaction (*F*(1, 96) = 0.74, *p* = .39, η_p_^2^ = 0.01). Although both accurate sources were correct 80% of the time, participants rated the similar-accurate source as more competent at the blap task (*M* = 75%, *SD* = 12.91) than the dissimilar-accurate source (*M* = 63%, *SD* = 18.83; comparison between the two *t*(96) = 5.52, *p* < .001, *d* = 0.72). Likewise, although both random sources were accurate only 50% of the time participants rated the similar-random source as more competent (*M* = 69%, *SD* = 17.24) than the dissimilar-random source (*M = 56%, SD = 20.26;* comparison between the two *t(96)* = *5.89, p < .001, d = 0.73;* Fig. 3B). Interestingly, the source that had different political views but excelled at the blap task (dissimilar-accurate) was rated less competent on the blap task than the source that performed randomly but was politically like-minded (*t*(96) = −2.58, *p* = .011, *d* = −0.33).

#### 2.2.3 An illusory perception of competence on shape task mediates the relationship between political similarity and information-seeking behavior

The above results suggest that political similarity influenced perceptions of source competence, with more politically similar sources viewed as more competent than their equally accurate counterparts. Does this explain the tendency to turn to politically like-minded people for information on blaps?

To test this possibility formally, we performed a causal mediation analysis (Fig. 3c) that asks whether the relationship between objective political similarity and information seeking behaviour is mediated by subjective ratings of competence on the blap task.

A multilevel modelling approach was used (Preacher, 2015), which allows for the appropriate treatment of non-independent observations by nesting trial-level observations within upper-level units (individual participants). Bayesian estimation of the multilevel mediation model was performed in the R programming language, using the open-source software package bmlm (Vuorre & Bolger, 2017). The bmlm package estimates regression models, with individual-level and group-level parameters estimated simultaneously using Markov chain Monte Carlo (MCMC) procedures. The default MCMC sampling procedure was employed, with 4 MCMC chains and 2000 iterations.

The mediation model examined whether perceived competence mediates the relationship between objective political similarity and source chosen with a predictor (X; source political similarity), mediator (M; competence rating), and dependent variable (Y; percentage each source was chosen). Indeed, we found a significant indirect effect of political similarity on choice through subjective competence rating (path ab: *M_posterior_* = 2.44, *SD* = 0.53, *CI* = [1.47, 3.54]).

The model shows the following. First, objective political similarity predicted how likely the participant was to turn to a source for information about blaps (total effect: *M_posterior_* = 6.10, *SD* = 1.05, *CI* = [4.06, 8.20]). Politically like-minded sources were, in general, chosen more often. This effect was attenuated, though not eliminated, when controlling for subjective competence ratings (path c′: *M_posterior_* = 3.66, *SD* = 1.04, *CI* = [1.63, 5.71]). Second, objective political similarity was positively related to subjective competence ratings (path a: *M_posterior_* = 6.32, *SD* = 0.94, *CI* = [4.45, 8.17]); similar sources were perceived as more competent. Third, subjective competence ratings predicted choice when objective political similarity was accounted for (path b: *M_posterior_* = 0.39, *SD* = 0.06, *CI* = [0.27, 0.50]), suggesting that subjective competence had a unique effect on choice.

#### 2.2.4 Accuracy on the blap task affects perception of similarity

The above results suggest that the effect of political similarity on participants’ choice of whom to turn to for information on blaps is partially mediated by their (illusory) subjective perception of the source’s competence on the blap task. One may ask, though, whether the reverse relationship is also true. Although less intuitive, could it be that sources that are more accurate on blaps are perceived to be more similar and that this perceived similarity mediates a relationship between objective accuracy and information seeking behaviour?

**Fig. 4:**
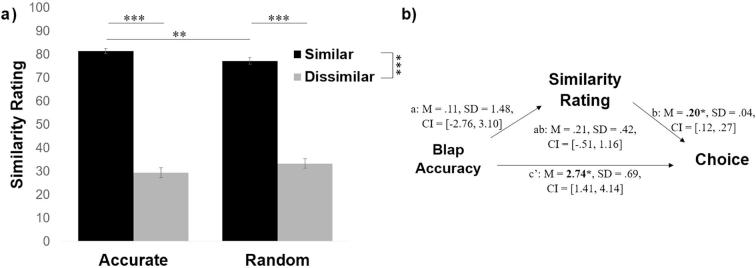
Accuracy on blap task partially enhances sense of similarity. (a) Politically similar sources were rated as more similar by participants. Interestingly the politically like-minded source that was also more accurate on blaps was rated as more similar than the politically like-minded source that was random on blaps. This suggests that accuracy on blap task partially affected perceived similarity. (b) The reverse mediation to that tested in Fig. 3 – by which perceived similarity mediates the effect between source accuracy and information seeking behaviour – was not significant. Error bars represent SEM. ^**^p < .01, ^***^p < .001.

To answer this question, we first examined how participants rated the sources on similarity. Entering similarity ratings into a 2 (similar/dissimilar) × 2 (accurate/random) ANOVA revealed a sensible main effect of political similarity (*F*(1, 96) = 648.76, *p* < .001, η_p_^2^ = 0.87) and no significant main effect of accuracy (*F*(1, 96) = 0.013, *p* = .91, η_p_^2^ < 0.01). An interaction also emerged (*F*(1, 96) = 7.23, *p* = .008, η_p_^2^ = 0.07). The interaction was due to the fact that while both politically similar sources agreed with the participant 80% of the time on political trials, there was an illusory perception that the more accurate source on blaps (similar-accurate) was significantly more similar to the subject (*M* = 81%, *SD* = 11.81) than the source that performed randomly on the blap task (similar-random, *M* = 77%, *SD* = 14.15, difference between the two: *t*(96) = 2.48, *p* = .015, *d* = 0.33). The two politically dissimilar sources were not rated as significantly different on similarity (dissimilar-accurate *M* = 29%, *SD* = 20.44; dissimilar-random *M* = 33%, *SD* = 20.03; comparison between the two t(96) = −1.50, *p* = .14, *d* = −0.19; Fig. 4a).

The above results reveal an illusion by which a source that is more accurate on the blap task is viewed as more similar to the self, perhaps revealing a motivation to associate the self with successful, similar others. We therefore conducted a second meditation analysis, using the same procedure as above, to examine whether perceived similarity mediates the relationship between objective accuracy and source chosen, with a predictor (X; source accuracy), mediator (M; similarity rating), and dependent variable (Y; percentage each source was chosen).

Our mediation model showed that it was not the case that subjective similarity mediated a relationship between objective accuracy on the blap task and information seeking behaviour (Fig. 4b). We did not find a significant effect of source accuracy on similarity rating nor did we find evidence of an indirect effect.

In particular, the mediation showed that objective accuracy on the blap task predicted how likely the participant was to turn to a source for information about blaps (total effect: *M_posterior_* = 2.96, *SD* = 0.81, *CI* = [1.42, 4.57]), showing that accurate sources were chosen more often. The effect was not, however, reduced when subjective similarity was controlled (path c′: *M_posterior_* = 2.74, *SD* = 0.69, *CI* = [1.41, 4.14]), suggesting that the accuracy-related variance in source choice is not shared with subjective similarity. Although subjective similarity ratings predicted choice when objective blap accuracy was accounted for (path b: *M_posterior_* = 0.20, *SD* = 0.04, *CI* = [0.12, 0.27]), suggesting that subjective similarity had a unique effect on choice, objective accuracy was not predictive of subjective similarity ratings (path a: *M_posterior_* = 0.11, *SD* = 1.48, *CI* = [−2.76, 3.10]), and the indirect effect of accuracy on the blap task on choice through subjective similarity rating was not significant (path ab: *M_posterior_* = 0.21, *SD* = 0.42, *CI* = [−0.51, 1.16]).

#### 2.2.5 Participants’ shape judgments are more *influenced* by sources that are politically like-minded

Thus far we find that participants are inclined to turn to sources that are like-minded politically to receive information on blaps. Are they also more likely to be influenced by them? We quantified the extent to which participants were influenced by a source by calculating the percentage of times the participant changed their answer when a source disagreed with them (only participants who chose to hear from each source at least once could be included in this analysis, N included = 70).

**Fig. 5:**
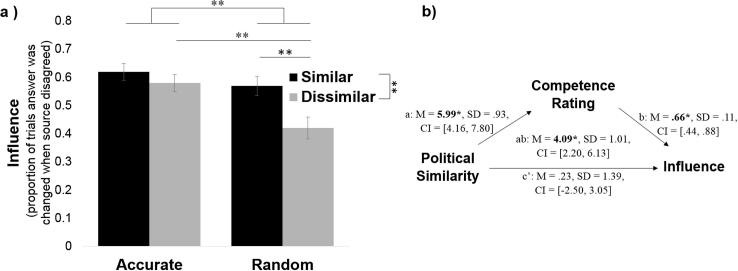
Participants’ blap judgments are more *influenced* by sources that are politically like-minded. (a) Participants were more likely to change their minds about blaps when sources that were (i) more accurate at the blap task and (ii) more politically like-minded disagreed with their blap judgment than when sources that were less accurate on blaps and/or politically different disagreed with their blap judgement. (b) A mediation model revealed that the relationship between political similarity and source influence was mediated by perceived competence on the blap task. Error bars represent SEM. ^**^p < .01.

We find that after choosing whom to listen to participants are more influenced by the sources that are politically like-minded and more accurate on the blap task (Fig. 5a). Participants changed their decisions on disagreement trials most often in response to information from the similar-accurate source (*M* = 62%, *SD* = 30.02; significantly greater than chance: t(93) = 4.74, *p* < .001) followed by the dissimilar-accurate source (*M* = 58%, *SD* = 29.59; significantly greater than chance: t(93) = 3.31, *p *= .001), followed by the similar-random source (*M* = 57%, *SD* = 29.94; significantly greater than chance: t(95) = 3.10, *p* = .003) and finally by the dissimilar-random source (*M* = 42%, *SD* = 34.72; not different from chance level: t(76) = −1.70, *p* = .093).

Entering percentage of answers changed on disagreement trials into a two (similar/dissimilar) by two (accurate/random) repeated-measures ANOVA revealed a main effect of source accuracy (*F*(1, 69) = 8.90, *p* = .004, η_p_^2^ = 0.11), a main effect of political similarity (*F*(1, 69) = 7.14, *p* = .009, η_p_^2^ = 0.09) and an interaction effect (*F*(1, 69) = 3.98, marginal *p* = .050, η_p_^2^ = 0.06). Post-hoc *t*-tests showed that the interaction was due to the accurate-dissimilar source having greater influence than the random-dissimilar source (t(73) = 3.24, *p *= .002, *d *= 0.53) while there was no difference in influence between the two similar sources (t(92) = 1.43, *p* = .16, *d* = 0.29). Similar results are obtained when incorporating both judgement and confidence into a measure of influence (see supplementary results).

Mean reaction times for participants’ final decisions were as follows: accurate-similar source (*M* = 1.16 s, *SD* = 2.06); accurate-dissimilar source (*M* = 1.42, *SD* = 10.56); random-similar source (*M* = 1.87, *SD* = 20.05), random-dissimilar source (*M* = 1.73, *SD* = 3.62). Entering log reaction times for participants’ decision to stick with or alter their choice into a 2 (similar/dissimilar) by 2 (accurate/random) ANOVA did not reveal any main effects or interaction (all P > .20).

The results suggest that both accuracy on the blap task and political similarity exert an effect on how influenced participants are by the sources. We next conducted a mediation model to test whether the effect of political similarity on influence was mediated by perceived accuracy on the blap task. Results of the multilevel mediation showed that objective political similarity predicted source influence (total effect: *M_posterior_* = 4.32, *SD* = 1.44, *CI* = [4.32, 1.54]) and was also positively related to the subjective ratings of competence (path a: *M_posterior_* = 5.99, *SD* = 0.93, *CI* = [4.16, 7.80]), which in turn predicted source influence when source similarity was accounted for (path b: *M_posterior_* = 0.66, *SD* = 0.11, *CI* = [0.44, 0.88]). The indirect effect of political similarity on source influence through competence rating was significant (path ab: *M_posterior_* = 4.09, *SD* = 1.01, *CI* = [2.20, 6.13]) and once subjective competence rating was controlled for political similarity no longer predicted source influence (path c′: *M_posterior_* = 0.23, *SD* = 1.39, *CI* = [−2.50, 3.05]). These results demonstrate that the effect of political similarity on influence is fully mediated by the perceived competence of the source (Fig. 5b).

Note that the conceptually reverse mediation model, with objective source accuracy as the predictor, subjective political similarity as the mediator and source influence as the dependent variable was not significant (no significant effect of objective accuracy on subjective similarity nor an indirect effect on source influence).

In particular, the model shows that objective accuracy on the blap task predicted source influence (total effect: *M_posterior_* = 4.34, *SD* = 1.37, *CI* = [1.65, 7.09]), showing that accurate sources had more influence. The effect was still significant when controlling for subjective similarity (path c′: *M_posterior_* = 4.56, *SD* = 1.35, *CI* = [1.91, 7.31]), suggesting that objective accuracy had a unique effect on source influence. Again, although subjective similarity ratings predicted source influence when objective blap accuracy was accounted for (path b: *M_posterior_* = 0.19, *SD* = 0.05, *CI* = [0.08, 0.30]), suggesting that subjective similarity had a unique effect on source influence, objective accuracy was not predictive of subjective similarity ratings (path a: *M_posterior_* = −1.39, *SD* = 1.53, *CI* = [−4.49, 1.62]), and the indirect effect of accuracy on the blap task on source influence through subjective similarity rating was not significant (path ab: *M_posterior_* = −0.22, *SD* = 0.38, *CI* = [−0.98, 0.54]).

The results of Experiment 1 suggested that knowledge of another’s political views interferes with the ability to learn about that person’s competence in an unrelated task. Politically like-minded sources were more likely to be chosen and the information they provided had a greater influence on participants’ decisions. Our mediation analyses suggest that participants preferred to hear from, and were more influenced by, politically similar sources because they falsely believed these sources were better at categorizing blaps than politically dissimilar sources.

## 3 Experiment 2

In Experiment 2 we test whether the findings of Experiment 1 replicate with minor adjustments to the methods (see below).

### 3.1 Methods

#### 3.1.1 Participants

The recruitment procedure was the same as for Experiment 1. In Experiment 2, 186 participants completed the Learning Stage. 101 (47 females and 54 males, aged 18–63 years *M* = 37.59, *SD* = 10.92) passed the learning test and proceeded to the Choice Stage.

Participants who passed the learning test did not differ from those who failed on age, gender, ethnicity, language, political ideology, interest/involvement in US politics, or generalized trust (all P > .18). Unlike in Experiment 1, participants that passed tended to have higher income (t(184) = 2.06, *p* = .041), education (t(184) = 2.59, *p* = .010) and subjective socio-economic-position (t(184) = 4.83, *p* < .001).

There was a strong positive correlation between performance on the attention check and accuracy on the learning test (r = 0.44, *p* < .001), suggesting that participants who passed the learning test (by answering at least eleven out of twelve trials correctly) were more attentive than those who failed. Participants who passed the learning test were correct on a greater number of the attention check questions (*M* = 92%, *SD* = 11.80) than those who failed the learning test (*M* = 78%, *SD* = 18.54; comparison between the two t(184) = 6.31, *p* < .001, *d* = 0.91). As in Experiment 1, participants who failed the learning test were not progressed to the choice stage and thus did not complete the main experimental task. In the choice stage, participants answered 74% of the attention checks correctly (SD = 19.71).

#### 3.1.2 Study design

The methods of Experiment 2 were the same as in Experiment 1 except for the following changes:

(i) Contrary to Experiment 1, we did not determine in advance which stimuli were blaps. Rather, feedback was given regardless of stimulus shown such that all participants were told they were correct on exactly 50% of the blap trials and incorrect on exactly 50% of blap trials. In contrast, in Experiment 1 participants’ accuracy rates depended on whether a stimulus was in fact coded to be a blap or not. Thus, accuracy rates differed across participants with an average of 48% (*SD* = 10.57).
(ii) The percentage of times the sources gave the same answer to the participant’s answer on *blap trials* was held constant at exactly 50% for each subject and source. In contrast, in Experiment 1 the percentage of times the sources gave the same answer as the participant on blap trials was not hard-coded and normally distributed around 50% (*SD* = 11.52).
(iii) The wording of one of the post-task questions was changed slightly to read “How politically similar do you think this source was to you?”
(iv) We added the following post-task question “How competent do you think you were at figuring out if each object was a blap?” 0 = “Very incompetent” to 100 = “Very competent”.
(v) Attention-check data was successfully recorded.

Changes 1–3 enables us to test for replication under slightly different conditions. Change 4 was to test for participants’ perception of their own ability.

### 3.2 Results

#### 3.2.1 Participants prefer to receive information about shapes from politically like-minded sources

**Fig. 6:**
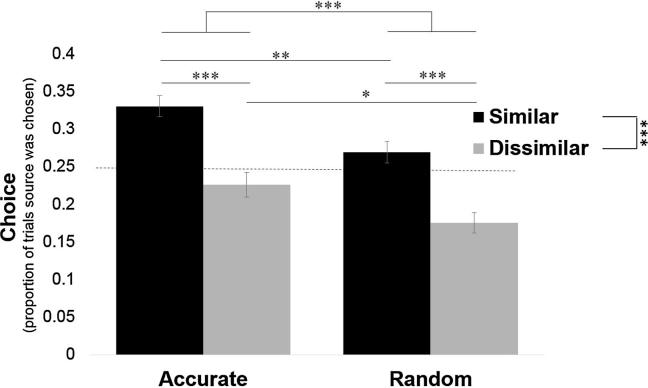
Participants prefer to receive information about shapes from politically like-minded sources. For each participant we calculated the percentage of times they selected to hear from each source about blaps out of all trials and averaged across participants. As each source was presented as an option an equal number of times if the participants had no preference each source would be selected about 25% of trials. A preference (main effect) for both accurate sources over inaccurate sources and for politically similar sources over politically dissimilar sources was found. Error bars represent SEM. ^*^p < .05, ^**^p < .01, ^***^p < .001.

As in Experiment 1, we find that participants sensibly prefer to hear from sources that are more accurate on the blap task, but also prefer to hear from politically like-minded sources even when they were not very good at the blap task (Fig. 6). Specifically, the similar-accurate source was chosen most often (*M* = 33%, *SD* = 14.18; significantly greater than chance: t(100) = 5.70, *p* < .001), followed by the similar-random source (*M* = 27%, *SD* = 14.16; not different from chance: t(100) = 1.32, *p* = .19), followed by the dissimilar-accurate source (*M* = 23%, *SD* = 16.72; not different from chance: t(100) = −1.45, *p* = .15) and finally by the dissimilar-random source (*M* = 18%, *SD* = 13.66; significantly lower than chance: t(100) = −5.51, *p* < .001). Entering the percentage of times each participant selected each source into a two (similar/dissimilar) by two (accurate/random) repeated-measures ANOVA revealed a main effect of source accuracy (*F*(1, 100) = 14.09, *p* < .001, η_p_^2^ = 0.12) and political similarity (*F*(1, 100) = 25.07, *p* < .001, η_p_^2^ = 0.20) with no interaction (*F*(1, 100) = 0.13, *p* = .72, η_p_^2^ = 0.001). Participants were not more likely to choose the source that was accurate on the blap task but dissimilar politically (dissimilar-accurate) over the politically like-minded source that performed randomly on the blap task (similar-random) (*t*(100) = −1.61, *p* = .11, *d* = −0.28).

Mean reaction times for source choice were as follows: accurate-similar source (*M* = 1.91, *SD* = 29.38); accurate-dissimilar source (*M* = 1.54 s, *SD* = 6.17); random-similar source (*M* = 1.59 s, *SD* = 5.94), inaccurate-dissimilar source (*M* = 1.53 s, *SD* = 6.28). Entering choice log reaction times into a 2 (similar/dissimilar) by 2 (accurate/random) ANOVA did not reveal any main effects or interaction (all P > .12).

#### 3.2.2 Political similarity leads to an illusory perception of competence on the shape task

**Fig. 7:**
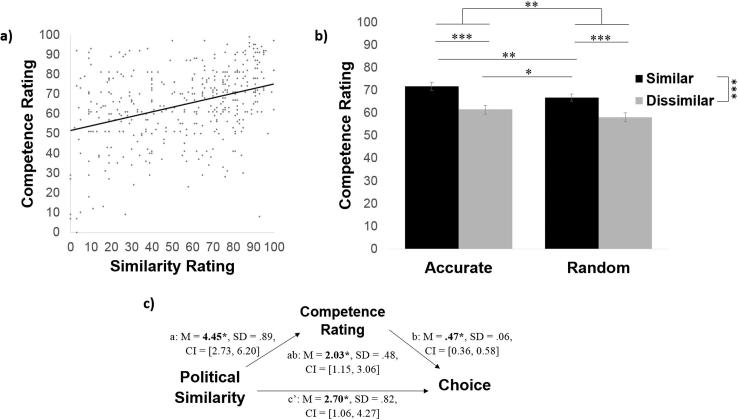
An illusory perception of accuracy mediates the relationship between political similarity and information seeking behavior. (a) The true correlation between how accurate a source was on the blap task and how like-minded they were to the participant was zero. Nevertheless, participants ratings revealed an illusory perception that the two were related (r = 0.36 *p* < .001). (b) Participants rated accurate sources as more competent on the blap task, but also rated politically like-minded sources as more competent on the blap task. (c) A mediation model revealed that perceived competence partially mediated the relationship between political similarity and choice of which source to hear from about blaps. Error bars represent SEM. ^*^p < .05,^**^p < .01, ^***^p < .001.

As in Experiment 1, we find that participants’ ratings of how similar the sources were to them correlated with their ratings of how competent they thought the sources were at the blap task. Specifically, participants had an *illusory* perception that the more similar the source was to them, the better the source was on the shape task (r = 0.36, *p* < .001, Fig. 7a).

We then assessed how participants rated the four sources on their ability to categorize blaps, entering these ratings into a two (similar/dissimilar) by two (accurate/random) repeated-measures ANOVA. This revealed a sensible main effect of source accuracy (*F*(1, 100) = 7.67, *p* = .007, η_p_^2^ = 0.07), an illusory main effect of source political similarity (*F*(1, 100) = 27.88, *p* < .001, η_p_^2^ = 0.22) and no interaction (*F*(1, 100) = 39, *p* = .53, η_p_^2^ < 0.01).

Participants rated the similar-accurate source as more competent at the blap task (*M* = 72%, *SD* = 17.12) than the dissimilar-accurate source (*M* = 62%, *SD* = 20.13; comparison between the two *t*(100) = 4.26, *p* < .001, *d* = 0.55). Likewise, participants rated the similar-random source as more competent (*M* = 67%, *SD* = 15.19) than the dissimilar-random source (*M = 58%, SD = 18.43;* comparison between the two t(100) = 4.18, *p* < .001, *d* = 0.51; Fig. 7**b**). The source that was politically like-minded but poor on the blap task (similar-random) was rated as more competent at the blap task than the source that performed well but had different political views (*t*(100) = −2.18, *p* = .031, *d* = −0.29).

#### 3.2.3 An illusory perception of competence on shape task mediates the relationship between political similarity and information-seeking behavior

We next test whether participants chose to hear from the politically similar sources because they believed they were more competent at the blap task. That is, we ask whether the relationship between objective political similarity and information seeking behaviour is mediated by subjective ratings of competence on the blap task. We used the same procedure as in Experiment 1 to perform this mediation analysis.

The model shows that objective political similarity predicted how likely the participant was to turn to a source for information about blaps (total effect: *M_posterior_* = 4.73, *SD* = 0.90, *CI* = [2.92, 6.46]), with politically like-minded sources chosen more often. This effect was attenuated, though not eliminated, when controlling for subjective competence ratings (path c′: *M_posterior_* = 2.70, *SD* = 0.82, *CI* = [1.06, 4.27]). Objective political similarity was positively related to subjective competence ratings (path a: *M_posterior_* = 4.45, *SD* = 0.89, *CI* = [2.73, 6.20]); similar sources were perceived as more competent. Subjective competence ratings predicted choice when objective political similarity was accounted for (path b: *M_posterior_* = 0.47, *SD* = 0.06, *CI* = [0.36, 0.58]), suggesting that subjective competence had a unique effect on choice. Finally, we find a significant indirect effect of political similarity on choice through subjective competence rating (path ab: *M_posterior_* = 2.03, *SD* = 0.48, *CI* = [1.15, 3.06]).

#### 3.2.4 Accuracy on the blap task affects perception of similarity

We next test whether sources that are more accurate on blaps are perceived as more similar and whether this increase in perceived similarity mediates the relationship between objective accuracy and information seeking behaviour.

We examined how participants rated the four sources on similarity, entering similarity ratings into a 2 (similar/dissimilar) × 2 (accurate/random) ANOVA. The results revealed a main effect of political similarity (*F*(1, 100) = 596.46, *p* < .001, η_p_^2^ = 0.86), no main effect of accuracy (*F*(1, 100) = 0.01, *p* = .94, η_p_^2^ < 0.01) and an interaction effect (*F*(1, 100) = 6.94, *p* = .010, η_p_^2^ = 0.07).

**Fig. 8:**
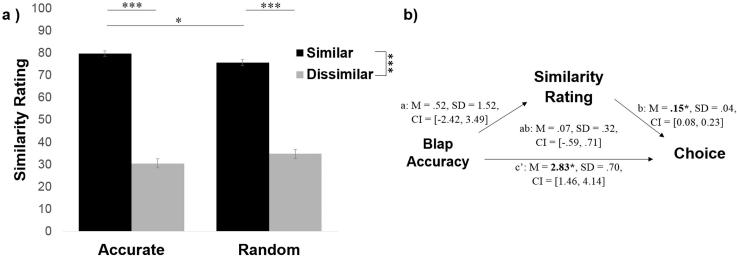
Accuracy on blap task partially enhances sense of similarity. (a) Politically similar sources were rated as more similar by participants. Interestingly the politically like-minded source that was also more accurate on blaps was rated as more similar than the politically like-minded source that was random on blaps. This suggests that accuracy on blap task partially affected perceived similarity. (b) The reverse mediation to that tested in Fig. 7c – by which perceived political similarity mediates the effect between source accuracy and information seeking behaviour – was not significant. Error bars represent SEM. ^*^p < .05, ^***^p < .001.

As in Experiment 1, for politically similar sources participants believed that the more accurate source on blaps (similar-accurate) was significantly more similar (*M* = 80%, *SD* = 12.24) than the source that performed randomly on the blap task (similar-random, *M* = 76%, *SD* = 14.57, difference between the two: *t*(100) = 2.30, *p* = .024, *d* = 0.30). The politically dissimilar sources were not rated as significantly different on similarity (dissimilar-accurate *M* = 30%, *SD* = 20.09; dissimilar-random *M* = 35%, *SD* = 20.69; comparison between the two *t*(100) = −1.60, *p* = .11, *d* = −0.21; Fig. 8). Thus our finding from Experiment 1 that sources that are both politically similar and accurate on the blap task are viewed as more similar to the self than sources that are politically similar but less accurate on the blap task was replicated.

We conducted another meditation analysis to examine whether perceived political similarity mediates the relationship between objective accuracy and source chosen, with a predictor (X; source accuracy), mediator (M; similarity rating), and dependent variable (Y; percentage each source was chosen).

Again, we did not find a significant effect of source accuracy on similarity rating nor did we find evidence of an indirect effect. The mediation showed that objective accuracy on the blap task predicted how likely the participant was to turn to a source for information about blaps (total effect: *M_posterior_* = 2.90, *SD* = 0.77, *CI* = [1.33, 4.40]), showing that accurate sources were chosen more often. The effect was not, however, reduced when subjective similarity was controlled (path c′: *M_posterior_* = 2.83, *SD* = 0.70, *CI* = [1.46, 4.14]), suggesting that the accuracy-related variance in source choice is not shared with subjective similarity. Although subjective similarity ratings predicted choice when objective blap accuracy was accounted for (path b: *M_posterior_* = 0.15, *SD* = 0.04, *CI* = [0.08, 0.23]), suggesting that subjective similarity had a unique effect on choice, objective accuracy was not predictive of subjective similarity ratings (path a: *M_posterior_* = 0.52, *SD* = 1.52, *CI* = [−2.42, 3.49]), and the indirect effect of accuracy on the blap task on choice through subjective similarity rating was not significant (path ab: *M_posterior_* = 0.07, *SD* = 0.32, *CI* = [−0.59, 0.71]).

#### 3.2.5 Participants’ shape judgments are more *influenced* by sources that are politically like-minded

**Fig. 9:**
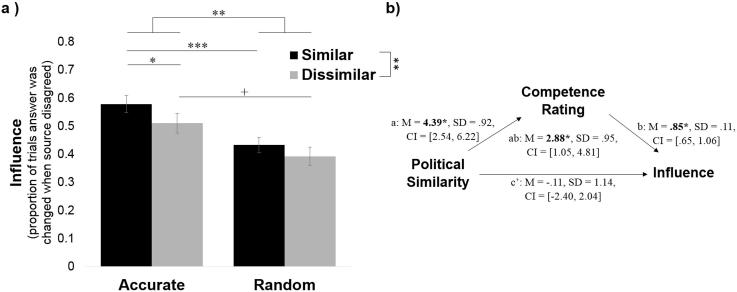
Participants’ blap judgments are more *influenced* by sources that are politically like-minded. (a) Participants were more likely to change their minds about blaps when sources that were (i) more accurate at the blap task and (ii) more politically like-minded disagreed with them than when sources that were random at the blap task or dissimilar disagreed with them. (b) A mediation model revealed that the relationship between political similarity and source influence was mediated by perceived accuracy on blap task. Error bars represent SEM. ^+^*p* < .10, ^*^p < .05, **p < .01, ^***^p < .001.

As in Experiment 1, participants were more influenced by the politically similar sources as well as those that are were more accurate on the blap task (N included = 75; Fig. 9a). Participants changed their answer most after hearing that the similar-accurate source disagreed with them (*M* = 58%, *SD* = 31.07; significantly different from chance: t(97) = 2.59, *p* = .011) followed by the dissimilar-accurate source (*M* = 52%, *SD* = 34.39; not different from chance: t(91) = 0.51, *p* = .61), followed by the similar-random source (*M* = 45%, *SD* = 27.59; not different from chance: t(94) = −1.73, *p* = .088) and finally by the dissimilar-random source (*M* = 41%, *SD* = 34.24; significantly lower than chance: t(92) = −2.43, *p* = .017).

Entering percentage of answers changed out of trials in which the source disagreed with the participants’ blap judgment into a two (similar/dissimilar) by two (accurate/random) repeated-measures ANOVA revealed a main effect of source accuracy (*F*(1, 74) = 11.27, *p* = .001, η_p_^2^ = 0.13), a main effect of political similarity (*F*(1, 74) = 7.36, *p* = .008, η_p_^2^ = 0.09) and no interaction (*F*(1, 74) = 0.72, *p* = .40, η_p_^2^ = 0.01). Similar findings are observed when measuring influence as a combination of judgement and confidence (see supplementary results).

Mean reaction times for participants’ final decisions were as follows: accurate-similar source (*M* = 1.22 s, *SD* = 3.61); accurate-dissimilar source (*M* = 1.30 s, *SD* = 8.67); random-similar source (*M* = 1.38 s, *SD* = 5.63), random-dissimilar source (*M* = 1.07 s, *SD* = 3.15). Entering log reaction times for participants’ decision to stick with or alter their choice into a 2 (similar/dissimilar) by 2 (accurate/random) ANOVA did not reveal any main effects or interaction (all P > .23).

We next conducted a mediation model to test whether the effect of political similarity on influence was mediated by perceived accuracy on the blap task. Results of the multilevel mediation showed that objective political similarity predicted source influence (total effect: *M_posterior_* = 2.77, *SD* = 1.32, *CI* = [0.15, 5.35]) and was also positively related to the subjective ratings of competence (path a: *M_posterior_* = 4.39, *SD* = 0.92, *CI *= [2.54, 6.22]), which in turn predicted source influence when source similarity was accounted for (path b: *M_posterior_* = 0.85, *SD* = 0.11, *CI* = [0.65, 1.06]). The indirect effect of political similarity on source influence through competence rating was significant (path ab: *M_posterior_* = 2.88, *SD* = 0.95, *CI* = [1.05, 4.81]) and once subjective competence rating was controlled for political similarity no longer predicted source influence (path c′: *M_posterior_* = −0.11, *SD* = 1.14, *CI* = [−2.40, 2.04]). These results demonstrate that the effect of political similarity on influence is fully mediated by the perceived competence of the source.

Note that the conceptually reverse mediation model, with objective source accuracy as the predictor, subjective political similarity as the mediator and source influence as the dependent variable was not significant (no significant effect of objective accuracy on subjective political similarity nor an indirect effect on source influence).

In particular, the model shows that objective accuracy on the blap task predicted source influence (total effect: *M_posterior_* = 5.32, *SD* = 1.22, *CI* = [2.90, 7.71]), showing that accurate sources had more influence. The effect was still significant when controlling for subjective similarity (path c′: *M_posterior_* = 5.25, *SD* = 1.20, *CI* = [2.86, 7.63]), suggesting that objective accuracy had a unique effect on source influence. Subjective similarity ratings did not predict source influence when objective blap accuracy was accounted for (path b: *M_posterior_* = 0.09, *SD* = 0.05, *CI* = [−0.01, 0.20]), objective accuracy was not predictive of subjective similarity ratings (path a: *M_posterior_* = 0.53, *SD* = 1.49, *CI* = [−2.42, 3.55]), and the indirect effect of accuracy on the blap task on source influence through subjective similarity rating was not significant (path ab: *M_posterior_* = 0.07, *SD* = 0.28, *CI* = [−0.49, 0.65]).

## 4 Discussion

The current study offers three central findings. The first is that people choose to hear from those who are politically like-minded on topics that have nothing to do with politics (like geometric shapes) in preference to those with greater expertise on the topic but have different political views. The second is that all else being equal, people are more influenced by politically like-minded others on nonpolitical issues such as shape categorization. The third is that people are biased to believe that others who share their political opinions are better at tasks that have nothing to do with politics, even when they have all the information they need to make an accurate assessment about who is the expert in the room. Our mediation analysis suggests that it is this illusion that underlies participants’ tendency to seek and use information from politically like-minded others.

A great deal of attention has recently been paid to what sources of political information people choose (Prior, 2007, Sunstein, 2017), how algorithms affect what they see (Garimella et al., 2018, Hannak et al., 2013, Sîrbu et al., 2018, Sunstein, 2017), and how people are affected by encountering diverse information on political issues (Colleoni et al., 2014, Druckman et al., 2013, Kahan, 2016, Tappin et al., 2017). There is also growing interest in how political affiliations affect people’s affective responses to those with different affiliations (Iyengar et al., 2012, Iyengar and Westwood, 2015).

Our focus here has been on epistemic spillovers -- on whether and how a sense of shared political convictions influences people’s desire to consult and to use people’s views on a task that is entirely unrelated to politics. The most striking finding is that people consult and are influenced by the judgments of those with shared political convictions even when they had observed evidence suggesting that those with different convictions are far more likely to offer the right answer.

While we manipulated similarity on political views, we hypothesize that similar findings may be observed when similarity is manipulated along other dimensions that are significant to people (e.g., music or literature preferences, hobbies etc.), a hypothesis that warrants empirical testing. Moreover, it would be of interest to test whether people are also more influenced by the like-minded when they receive information from sources passively and not actively (as in our study).

What accounts for our findings? We have referred to the halo effect: If people think that products or people are good along some dimension, they tend to think that they are good along other dimensions as well (Dion et al., 1972, Nisbett and Wilson, 1977, Thorndike, 1920). If people have an automatic preference for those who share their political convictions, their positive feelings may spill over into evaluation of other, unrelated characteristics (including their ability to identify blaps). This is one consequence of political tribalism.

A related explanation involves a heuristic, or mental shortcut, which often works well, but which can lead to severe and systematic errors (Kahneman & Frederick, 2002): If people generally believe that politically like-minded people are particularly worth consulting, they might extend that belief to contexts in which the belief does not make much sense. It is possible to take our findings here as evidence that people regularly use a heuristic of this kind and thus give special weight to the conclusions of those with similar political convictions, even when they know, or have reason to know, that those conclusions do not deserve that weight.

Our findings have implications for the spread of false news, for political polarization, and for social divisions more generally. A great deal of false news is political (Kuklinski et al., 2000, Kull et al., 2003) and it is spread by and among like-minded people (Del Vicario et al., 2016). But our findings suggest that among the politically like-minded, false news will spread even if it has little or nothing to do with politics, or even if the connection to politics is indirect and elusive. Suppose, for example, that someone with congenial political convictions spreads a rumor about a coming collapse in the stock market, a new product that supposedly cures cancer or baldness, cheating in sports, an incipient epidemic, or a celebrity who has shown some terrible moral failure. Even if the rumor is false, and even if those who hear it have reason to believe that it is false, they may well find it credible (and perhaps spread it).

The results help identify both a cause and a consequence of political polarization. If people trust like-minded others not only on political questions (Nyhan & Reifler, 2010) but also on questions that have nothing at all to do with politics, the conditions are ripe for sharp social divisions, potentially leading people to live in different epistemic universes.
