# Supplementary material for: Epistemic spillovers: Learning others’ political views reduces the ability to assess and use their expertise in nonpolitical domains
Source: Cognition. 2019 Jul;188:74–84. doi: 10.1016/j.cognition.2018.10.003 (PMC6522687; doi:10.1016/j.cognition.2018.10.003)
Supplement: Supplementary data 1 [file mmc1.docx]

**Supplementary Materials**

**Demographic Information (participants whom completed the entire experiment)**

**Experiment 1**

Participants reported:

Gender and age (descriptive statistics reported in main text).

Ethnicity: 78% White, 6% Black, 6% Hispanic, 7% Asian, 2% Other.

Whether English was their first language: 98% said “yes”, “2%” said no.

Highest level of education completed:

| High School Diploma | 37% |
| --- | --- |
| 2 Year Degree | 22% |
| 4 Year Degree | 33% |
| Postgraduate/Professional Degree | 7% |
| Other | 1% |

The approximate amount of income they earned in 2016:

| Under $5,000 | 11% |
| --- | --- |
| $5,000-$10,000 | 8% |
| $10,001-$15,000 | 6% |
| $15,001-$25,000 | 7% |
| $25,001-$35,000 | 16% |
| $35,001-$50,000 | 22% |
| $50,001-$65,000 | 8% |
| $65,001-$80,000 | 10% |
| $80,001-$100,000 | 6% |
| Over $100,000 | 5% |

Subjective socio-economic position on a 10-point scale (from 1 = “Worst off” to 10 = “Best off”; *M* = 6.54, *SD* = 1.62, Range = 3-10). A one-sample t-test showed that the mean was significantly different from the midpoint of the scale (*t*(96) = 9.34, *p* < .001), with participants reporting higher than average subjective socio-economic position.

Political ideology (on a sliding scale from 0 = “Liberal” to 1 = “Conservative”; *M* = .41, *SD* = .29, Range = 0-1). A one-sample t-test showed that the mean was significantly different from the 0.5 midpoint of the scale (*t*(96) = -2.99, *p* = .004), suggesting our sample was ideologically left of centre.

Interest/involvement in US politics (from 0 = “Not at all” to 100 = “Completely”; *M* = 60.96, *SD* = 27.40, Range = 0-100). A one-sample t-test showed that the mean was significantly different from the 50% midpoint of the scale (*t*(96) = 3.94, *p* < .001), with participants reporting greater interest and involvement than not.

Trust in other people that they interact with in daily life (from 1 = “Very little” to 7 = “Very much”; *M* = 4.92, *SD* = 1.60, Range = 1-7). A one-sample t-test showed that the mean was significantly different from the midpoint of the scale (*t*(96) = 8.73, *p* < .001), with participants reporting high levels of trust in the people they interact with.

**Experiment 2**

Gender and age (descriptive statistics reported in main text).

Ethnicity: 81% White, 5% Black, 5% Hispanic, 9% Asian, 0% Other.

English was first language: 99% said “yes”, “1%” said no.

Highest level of education completed:

| High School Diploma | 29% |
| --- | --- |
| 2 Year Degree | 25% |
| 4 Year Degree | 34% |
| Postgraduate/Professional Degree | 13% |
| Other | 0% |

The approximate amount of income they earned in 2016:

| Under $5,000 | 11% |
| --- | --- |
| $5,000-$10,000 | 8% |
| $10,001-$15,000 | 5% |
| $15,001-$25,000 | 20% |
| $25,001-$35,000 | 8% |
| $35,001-$50,000 | 13% |
| $50,001-$65,000 | 18% |
| $65,001-$80,000 | 11% |
| $80,001-$100,000 | 4% |
| Over $100,000 | 3% |

Subjective socio-economic position on a 10-point scale (from 1 = “Worst off” to 10 = “Best off”; *M* = 6.49, *SD* = 1.76, Range = 1-10). A one-sample t-test showed that the mean was significantly different from the midpoint of the scale (*t*(100) = 8.46, *p* < .001), with participants reporting higher than average subjective socio-economic position.

Political ideology (on a sliding scale from 0 = “Liberal” to 1 = “Conservative”; *M* = .45, *SD* = .32, Range = 0-1). A one-sample t-test showed that the mean was not significantly different from the midpoint of the scale (*t*(100) = -1.64, *p* = .11).

Interest/involvement in US politics (from 0 = “Not at all” to 100 = “Completely”; *M* = 66.91, *SD* = 26.24, Range = 0-100). A one-sample t-test showed that the mean was significantly different from the midpoint of the scale (*t*(100) = 6.48, *p* < .001), with participants reporting higher than average interest and involvement in politics.

Trust in other people that they interact with in daily life (from 1 = “Very little” to 7 = “Very much”; *M* = 5.04, *SD* = 1.47, Range = 1-7). A one-sample t-test showed that the mean was significantly different from the midpoint of the scale (*t*(100) = 10.53, *p* < .001), with participants reporting high levels of trust in the people they interact with.

**Debrief Questions**

**Experiment 1**

In the debrief, participants were asked:

To report what they thought was the purpose of the study in an open-answer format: 57 participants reported that they did not know what the purpose of the study was or provided an incorrect answer (e.g. “To determine memory recall”). 40 participants provided answers that were related to a goal or sub-goal of the study (i.e. answers that mentioned testing relationship between similarity, influence and/or competence).

Whether they found any rule(s) to decide if each object was a blap, and what the rules were: 51 participants reported a rule, 44 reported that they did not know, one said it seemed random, and one did not answer the question.

How sure they were that their rule(s) were correct (from 0 = Not at all confident, to 100 = Very confident; *M* = 39.38, *SD* = 26.72, Range = 0-100). A one-sample t-test showed that the mean was significantly different from the midpoint of the scale (*t*(96) = -3.91, *p* < .001), with participants reporting low levels of confidence in their rule(s) for categorising blaps.

How many blaps there were in the task, as a percentage (*M* = 59.80, *SD* = 17.51, Range = 5-95). A one-sample t-test showed that the mean was significantly different from the midpoint of the scale (*t*(96) = 5.52, *p* < .001), with participants believing that a greater than average percentage of the items in the task were blaps.

How well they learned about the accuracy of the four sources in the blap task: 24 participants thought they learned about the accuracy of all four, 66 thought they learned about some of the sources, 4 thought they didn’t learn about any of the sources and 3 reported that they did not know.

How well they learned about the political opinions of the four sources: 37 participants thought they learned about all four, 49 thought they learned about some of the sources, 7 thought they didn’t learn about any of the sources and 4 reported that they did not know.

To rate each source on a number of dimensions:

|  | **Similar-Accurate** | **Dissimilar-Accurate** | **Similar-Random** | **Dissimilar- Random** |
| --- | --- | --- | --- | --- |
| Competence in the blap task (see main text) | 71.93 (19.26) | 64.11 (23.58) | 67.54 (18.49) | 53.40 (23.09) |
| Consistency of performance in the blap task | 73.47 (18.92) | 64.11 (21.90) | 69.46 (15.98) | 56.45 (22.14) |
| Political views (from 0 = “Liberal” to 1 = “Conservative”) | .44 (.30) | .55 (.33) | .49 (.27) | .54 (.27) |
| Consistency of political views | 77.64 (16.11) | 67.78 (25.61) | 74.42 (16.59) | 62.11 (22.78) |
| Trust in source | 73.34 (19.53) | 54.75 (25.18) | 67.94 (20.72) | 46.05 (24.75) |
| Similarity (see main text) | 77.06 (14.88) | 30.19 (21.54) | 70.60 (18.04) | 35.07 (22.90) |

Numbers presented in the table are means with standard deviations in parentheses

General impressions and any other comments for each source in open-answer format: the open-text answers tended to match up with the quantitative measurements.

Which source they preferred for blap questions: 42 participants reported they preferred the Similar-Accurate source, 25 the Dissimilar-Accurate, 23 the Similar-Random, 7 the Dissimilar-Random.

Which source they avoided for blap questions: 8 participants reported that they avoided the Similar-Random, 15 the Similar-Accurate source, 19 the Dissimilar-Accurate, 55 the Dissimilar-Random.

How they made decisions about which source to choose for blap questions in open-answer format: participants generally reported that they chose the source that seemingly performed the best in the learning stage.

Whether they chose a source that they thought would be wrong so that they could do the opposite: 15 participants reported that they used this strategy.

To what extent they believed that the responses from the sources were those of previous participants?” (from 0 = “Did not at all believe it” to 100 = “Completely believed it”). Despite the question being the last in the funneled debriefing, and thus the most specific and closed-ended, the ratings revealed only mild suspicion rates with a mean score not significantly different from the mid-point of the scale (*M* = 44.44, *SD* = 33.96, *t*(96) = -1.61, *p* = .11).

Finally, participants were thanked and asked if any of the instructions were unclear and if they had any final comments for the researchers.

**Experiment 2**

Participants reported:

What they thought was the purpose of the study in an open-answer format: 72 participants reported that they did not know what the purpose of the study was or provided an incorrect answer. 29 participants provided answers that were related to a goal or sub-goal of the study (i.e. answers that mentioned testing relationships between similarity, influence and/or competence).

Whether they found any rule(s) to decide if each object was a blap, and what the rules were: 52 participants reported a rule, 35 reported that they did not know, 3 said it seemed random, 8 said that they tried to remember specific examples and 6 provided answers that did not address the question.

How sure they were that their rule(s) were correct (from 0 = Not at all confident, to 100 = Very confident; *M* = 41.09, *SD* = 26.53, Range = 0-100). A one-sample t-test showed that the mean was significantly different from the midpoint of the scale (*t*(100) = -3.38, *p* < .001), with participants reporting low levels of confidence in their rule(s) for categorising blaps.

How many blaps there were in the task, as a percentage (*M* = 58.52, *SD* = 18.08, Range = 10-100). A one-sample t-test showed that the mean was significantly different from the midpoint of the scale (*t*(100) = 4.74, *p* < .001), with participants believing that a greater than average percentage of the items in the task were blaps.

How competent they thought they were at the blap task (from 0 = Very incompetent, to 100 = Very competent; *M* = 44.78, *SD* = 23.16, Range = 0-100). A one-sample t-test showed that the mean was significantly different from the midpoint of the scale (*t*(100) = -2.26, *p* = .026), with participants believing they were worse than average at guessing which shapes were blaps.

How well they learned about the accuracy of the four sources in the blap task: 26 participants thought they learned about the accuracy of all four, 61 thought they learned about some of the sources, 8 thought they didn’t learn about any of the sources and 6 reported that they did not know.

How well they learned about the political opinions of the four sources: 36 participants thought they learned about all four, 58 thought they learned about some of the sources, 3 thought they didn’t learn about any of the sources and 4 reported that they did not know.

To rate each source on a number of dimensions:

|  | **Similar-Accurate** | **Dissimilar-Accurate** | **Similar-Random** | **Dissimilar- Random** |
| --- | --- | --- | --- | --- |
| Competence in the blap task (see main text) | 67.80 (20.01) | 62.55 (20.25) | 61.48 (18.79) | 56.67 (20.04) |
| Consistency of performance in the blap task | 68.50 (18.51) | 62.03 (20.58) | 61.92 (17.63) | 59.40 (18.25) |
| Political views (from 0 = “Liberal” to 1 = “Conservative”) | .50 (.28) | .52 (.33) | .46 (.28) | .55 (.28) |
| Consistency of political views | 72.09 (15.10) | 64.55 (21.52) | 69.03 (17.84) | 62.71 (19.74) |
| Trust in source | 69.05 (21.27) | 52.83 (26.20) | 61.25 (20.35) | 50.09 (23.77) |
| Similarity (see main text) | 71.99 (16.84) | 29.98 (22.53) | 66.89 (21.79) | 34.74 (21.26) |

Numbers presented in the table are means with standard deviations in parentheses

General impressions and any other comments for each source in open-answer format: the open-text answers tended to match up with the quantitative measurements.

Which source they preferred for blap questions: 41 participants reported they preferred the Similar-Accurate source, 24 the Dissimilar-Accurate, 24 the Similar-Random, 12 the Dissimilar-Random.

Which source they avoided for blap questions: 11 participants reported that they avoided the Similar-Random, 15 the Similar-Accurate source, 37 the Dissimilar-Accurate, 38 the Dissimilar-Random.

How they made decisions about which source to choose for blap questions in open-answer format: participants generally reported that they chose the source that seemingly performed the best in the learning stage.

Whether they chose a source that they thought would be wrong so that they could do the opposite: 28 participants reported that they used this strategy.

To what extent they believed that the responses from the sources were those of previous participants?” (from 0 = “Did not at all believe it” to 100 = “Completely believed it”). Despite the question being the last in the funneled debriefing, and thus the most specific and closed-ended, the ratings revealed only mild suspicion rates with a mean score not significantly different from the mid-point of the scale (*M* = 45.58, *SD* = 30.18, *t*(100) = -1.47 *p* = .15).

Finally, participants were thanked and asked if any of the instructions were unclear and if they had any final comments for the researchers.

**Political Statement Stimuli**

The political statement stimuli were adapted from the following sources:

[https://www.isidewith.com/political-quiz#](https://www.isidewith.com/political-quiz)
<https://www.isidewith.com/polls>
<http://www.people-press.org/quiz/political-party-quiz/>

Participants saw the following four practice stimuli and 80 statements from the Learning Stage stimuli:

| **Practice Stimuli** |
| --- |
| Increasing gun laws and regulations would not deter crime in the USA |
| Lowering the minimum voting age would help get young people interested in politics |
| A politician formerly convicted of a crime would likely make bad decisions in office |
| Remaining in NATO will help secure a peaceful future for the USA |
|  |
| **Learning Stage Stimuli** |
| The risks from offshore oil drilling are minimal |
| The Paris Climate Agreement disadvantages US businesses and workers |
| Assassinating suspected terrorists in foreign countries helps keep the world a safe place |
| Deporting immigrants who are potential threats will make America safer |
| A stricter USA immigration policy will improve social cohesion |
| Americans will flourish if immigrants who commit crimes are deported |
| Immigrants would fit in better if compelled to learn English |
| America could improve national safety with tighter border control |
| Immigrants take jobs away from people born in the USA |
| High immigration results in lower wages for US citizens |
| Illegal immigrants need to feel scared of being deported to prevent more coming to the USA |
| Immigrants abuse the welfare system |
| Women who get abortions usually don't understand the consequences of what they are doing |
| Gay marriage confuses children |
| Nuclear power is an unsafe method for generating energy |
| Cutting public spending will reduce national debt |
| Lowering the tax rate for corporations will reduce unemployment in America |
| Spending less on social welfare will motivate people to work |
| Low restrictions on access to welfare benefits encourages people to abuse the system |
| Welfare recipients usually spend the money on drugs and alcohol |
| Labour unions hurt the economy |
| Ordinary people get a good proportion of the nation’s wealth |
| Encouraging private enterprise will improve the US economy |
| Private corporations educate political parties about important issues through lobbying |
| Assassinating suspected terrorists in foreign countries helps keep America safe |
| Terrorism would decrease if government surveillance were expanded to combat terrorism |
| Increased spending on the military will help to keep America safe |
| Allowing the police to monitor the phone calls and emails of criminals helps keep America safe |
| Giving nonviolent drug offenders mandatory jail sentences would reduce rates of delinquency |
| The bans on medicinal and recreational drugs protect people from themselves and others |
| Fracking is the safest way to keep oil prices low |
| Many of the claims about environmental threats are exaggerated |
| The death penalty deters people from committing crimes |
| The police would be more effective if they could access individual's private data |
| A worldwide American military presence helps maintain peace |
| A tough justice system keeps the crime rate low |
| Strong trade unions prevent industry goals from being achieved |
| Benefits for unemployed people are too high and discourage them from finding jobs |
| Most unemployed people don't try that hard to find a job |
| Patriotism improves social cohesion |
| Reducing civil liberties helps to maintain order in society |
| Newer lifestyles are contributing to a breakdown in society |
| Going to war is sometimes the only solution to international problems |
| Individual initiative needs to be incentivised to promote competition, even if this increases inequality |
| Criticising your country has an effect on your American identity |
| Society works better if people adhere to a simple unbending moral code |
| Society works better if all people are left to accomplish things on their own |
| Swift and severe punishment for criminals helps to maintain peace |
| Western civilization has brought more progress than all other cultural traditions |
| Social charities create dependency |
| Going to war with a country can actually improve outcomes for that country |
| Rewarding some more than others motivates competition |
| The police would be more effective if there were not so many rules preventing them from doing their jobs |
| Immigrants who work hard tend to find success in America |
| Building a wall around the southern border would reduce illegal immigration |
| It is unsafe to let Muslim immigrants enter the country until the government improves its ability to screen out potential terrorists |
| Illegal immigrants should not have access to government-subsidized healthcare |
| Health insurers exploit individuals who have a pre-existing medical condition |
| Requiring a photo ID before letting people vote would greatly impact election results |
| The death penalty is more than just a political tool |
| Internet service providers speed up access to popular websites (that pay higher rates) at the expense of slowing down access to other websites |
| Burning the American flag indicates a person may want to harm Americans |
| Privatization of veterans' healthcare will reduce the burden on society |
| The vast majority of offshore investing is perfectly legal |
| Forcing 18 year olds to provide at least one year of military service would make America safer |
| Formally declaring war on ISIS would make America safer |
| Local police could increase safety by increasing surveillance and patrol of Muslim neighborhoods |
| The military fly drones over foreign countries to gain intelligence and kill suspected terrorists |
| Markets suffer as a result of government interference |
| Allowing people who are against democracy to run in elections is a threat to democratic rights |
| The government's funding of planned parenthood improves child outcomes |
| Foreign Aid spending reduces worldwide suffering |
| The US did not decrease foreign aid spending during the last recession |
| Immigration gives a boost to the national economy |
| Immigrants help the US learn about beneficial new ideas |
| Homosexual couples have the same adoption rights as same sex couples |
| Businesses are generally more profitable if they have at least one woman on their board of directors |
| Listening to hate speech fuels extremist behavioural tendencies |
| Marijuana legalisation reduces violent crime |
| A diverse society is more creative than a homogeneous society |
| The US could increase national happiness by raising taxes on the rich |
| The government is legally obliged to ensure everyone is provided for |
| Providing everyone with a guaranteed basic income would reduce unhappiness in America overall |
| It is the government’s role to redistribute income to curb inequality |
| Human rights are often overridden to maintain national security |
| Forcing businesses to reduce carbon emissions will help protect the environment from climate change |
| The government do not focus on improving animal rights |
| The government do not use taxes to protect the environment |
| Economic growth typically harms the environment |
| Increasing government spending on public transport helps individuals and businesses to make money |
| Allowing convicted criminals the right to vote improves election outcomes |
| Releasing non-violent criminals from jail is only a small threat and reduces overcrowding |
| Zero-hour employment contracts are detrimental to workers |
| The national minimum wage prevents businesses exploiting workers |
| The national living wage is increased by more than inflation annually |
| Incomes are less equally distributed than Americans think |
| Supporting minimum income rising more sharply than other income levels would help to reduce crime |
| It is the government’s responsibility to provide a job for everyone who wants one |
| Big business tends to benefit owners at the expense of workers |
| Inequality reduction would improve social cohesion in America |
| The poor are discriminated against by the police |
| The police use racial stereotyping |
| Traditional values stop people from embracing technological advancements |
| Providing free health care to all citizens would bankrupt America |
| Individuals have the legal freedom to believe whatever they want |
| Every assertion made by our highest political and military leaders is subject to scrutiny |
| Treating other countries as equals makes them more willing to cooperate |
| It is harder for ethnic minorities to pass a job interview in America than it is for caucasians |
| Women are disadvantaged in the workplace compared to men |
| Giving disabled children extra resources at school will help them to overcome their handicaps |
| Disabled people should not face disadvantage in the workplace |
| Adding "Gender Identity" to anti-discrimination laws will reduce discrimination |
| Increasing restrictions on the current process of purchasing a gun would reduce gun crimes |
| Obamacare helps to provide affordable medical care to those who would not receive it otherwise |
| Reducing interest rates on student loans would increase the USA's intellectual dominance |
| Accepting refugees from Syria does not pose any great threat to America |
| Foreign terrorism suspects should be given constitutional rights |
| Removing confederate monuments and memorials from public grounds will reduce feelings of discrimination |
| Providing 'trigger warnings' and 'safe spaces' for students will increase their focus on learning rather than worrying about threats |
| Increasing the spending on public transportation will solve transportation problems, like traffic jams |
| A state displaying the confederate flag on government property is a sign of racism and seperatism |
| Banning a Niqab, or face veil, at civic ceremonies infringes on individual rights and prevents people from expressing their religious beliefs |
| The diversity due to affirmative action programs will give rise to innovation |
| When political candidates release their recent tax returns to the public, this increases their transparency |
| Increasing funding of health care for low income individuals would have a positive economic impact overall |
| Raising the tax rate for corporations will encourage companies to move to places with lower taxes |
| The Dakota Access pipeline will help the economy |
| Stricter smoking regulations will reduce the number of young people who start smoking |
| Allowing terminally ill patients to end their lives through assisted suicide will reduce unhappiness |
| The government should firmly control prices after wage increases |
| Positive discrimination is necessary to create a balance in the workplace and society |
| Abolishing the inheritance tax would result in increased inequality |
| Bringing essential public services and industries into state ownership would stop monopolies exploiting the public |
| Passing laws to protect whistle blowers would lead more people to come forward about wrong-doings |
| Increasing government spending will give a boost to the economy that is worth the extra debt |
| The government should regulate the price of life-saving drugs |
| Allowing the federal government to negotiate drug prices for Medicare will reduce health care costs |
| Making sure that everyone has an equal opportunity to succeed, will increase prosperity overall |
| Those with more resources have more obligations toward their fellow human beings |
| Abolishing the electoral college will lead to fairer elections |

**Additional Analyses**

**Post-Choice Task Similarity and Competence Ratings**

Ratings of subjective similarity and competence were elicited again at the very end of the study to check if the manipulation during the learning phase still affected participants’ evaluation of the sources. The results showed that it did.

In Experiment 1 entering post-choice similarity ratings into a two (similar/dissimilar) by two (accurate/random) repeated-measures ANOVA revealed a main effect of source political similarity (*F*(1,96) = 303.10, *p* < .001, ηp^2^ = .76), no main effect of source accuracy (*F*(1,96) = .17, *p* = .68, ηp^2^ = .002) and an interaction (*F*(1,96) = 10.81, *p* = .001, ηp^2^ = .10). Entering competence ratings into a two (similar/dissimilar) by two (accurate/random) repeated-measures ANOVA revealed a main effect of source accuracy (*F*(1,96) = 14.10, *p* < .001, ηp^2^ = .13), a main effect of source political similarity (*F*(1,96) = 20.00, *p* < .001, ηp^2^ = .17) and no interaction (*F*(1,96) = 3.23, *p* = .075, ηp^2^ = .03).

**
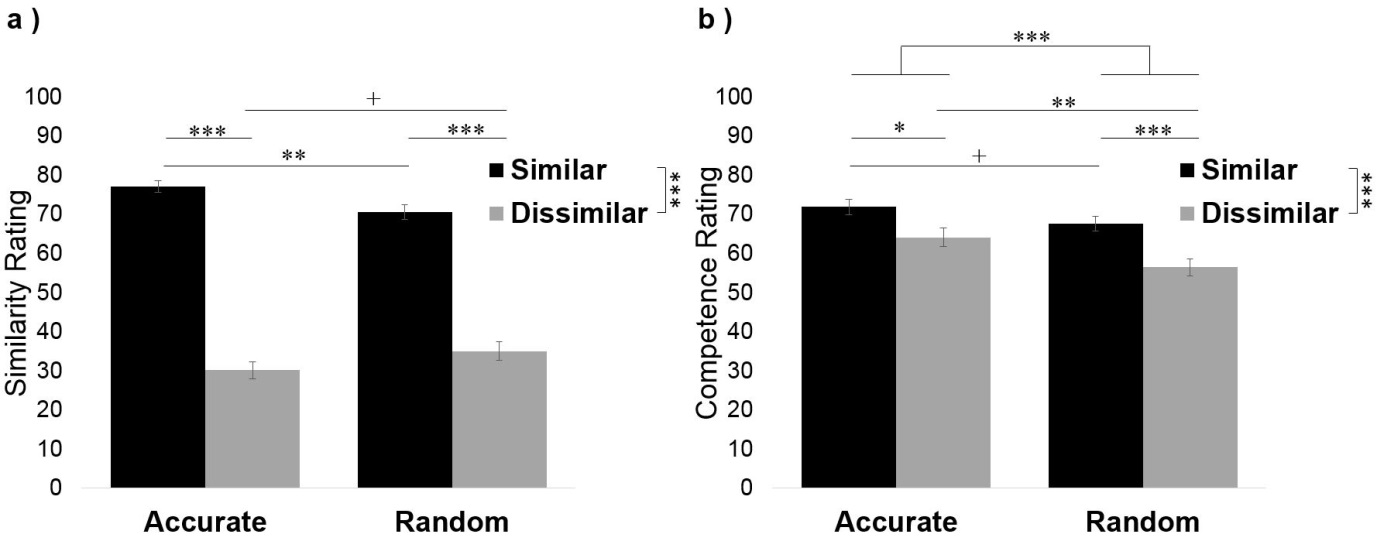
**

**Figure S1**. Subjective post-choice task (a) similarity ratings and (b) competence ratings of each source. Error bars represent SEM. ^+^*p*<.10, **p*<.05, ***p*<.01, ****p*<.001.

In Experiment 2 the wording of the post-task similarity rating question was changed slightly to check that participants construed this question as referring to politically similarity. Here participants specifically rated how *politically* similar the sources were to them (“How politically similar do you think this source was to you?” from 0 = “Not at all like me” to 100 = “Exactly like me”). Altering the wording of this question did not affect the results. In particular, entering the similarity ratings into a two (similar/dissimilar) by two (accurate/random) repeated-measures ANOVA revealed a main effect of source political similarity (*F*(1,100) = 234.39, *p* < .001, η**_p_**^2^ = .70), no main effect of source accuracy (*F*(1,100) = .01, *p* = .93, η**_p_**^2^ < .01) and an interaction (*F*(1,100) = 7.06, *p* = .009, η**_p_**^2^ = .07). The interaction effect replicated the pattern of results reported in the main text. Entering the competence ratings into a two (similar/dissimilar) by two (accurate/random) repeated-measures ANOVA revealed a main effect of source accuracy (*F*(1,100) = 10.85, *p* = .001, η**_p_**^2^ = .10), a main effect of source political similarity (*F*(1,100) = 5.29, *p* = .023, η**_p_**^2^ = .05) and no interaction (*F*(1,100) = .02, *p* = .90, η**_p_**^2^ < .01).


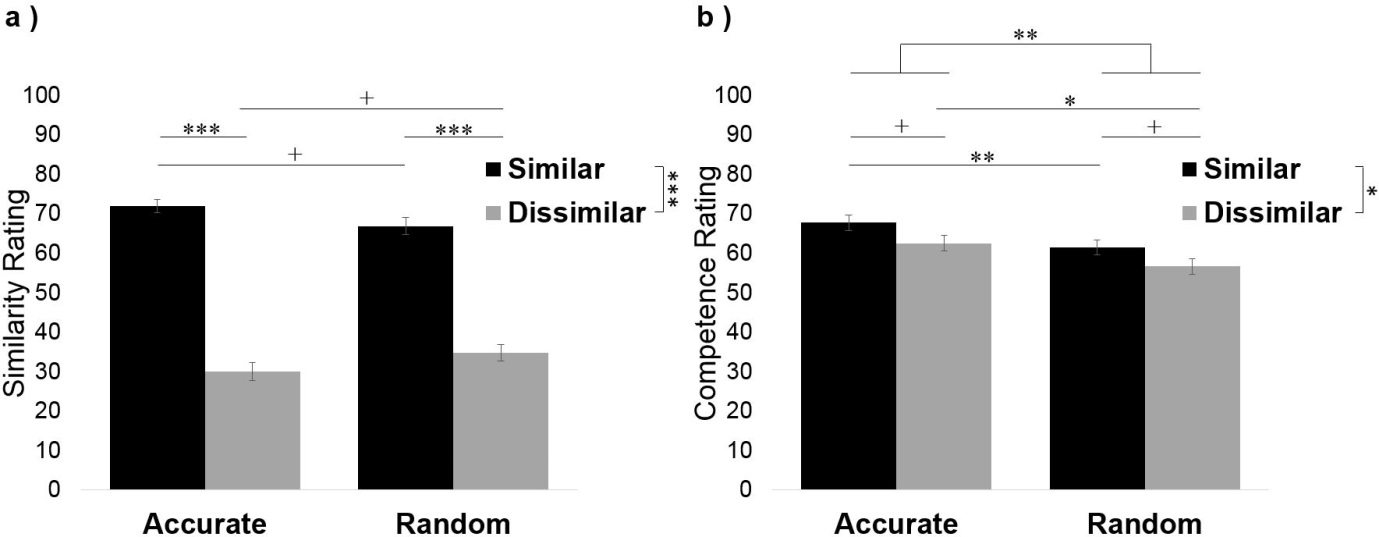


**Figure S2**. Subjective post-choice task (a) similarity ratings and (b) competence ratings of each source. Error bars represent SEM. ^+^*p*<.10, **p*<.05, ***p*<.01, ****p*<.001.

**Change of Mind (COM)**

In the Choice Stage participants rated how confident they were in both their initial answer and final answer. It was therefore possible to assess source influence based not only on the participant’s decision to keep or change their answer in response to new information but also according to how much confidence in the initial judgement was affected.

To incorporate confidence ratings we used the *Change of Mind* (COM) measure developed by our lab previously (Edelson, Dudai, Dolan, & Sharot, 2014). The COM measure takes into account (i) whether participants stick with their judgment or alter it when receiving information and (ii) the amount and direction by which they update their confidence.

The advantage of COM is that it detects differences in influence even when choices do not vary. Judgements can vary from the extreme case of believing X with high confidence (in this instance, believing a shape is a blap) to believing the opposite of X (that a shape is not a blap) with high confidence. In this experiment COM measures how judgements move along this axis after the participant sees the source’s response, with higher scores indicating greater source influence.

To illustrate this more concretely, let us imagine a participant believes a shape is a blap and provides a medium confidence of 50 on a scale from 0 to 100. Now let us imagine the source agrees – it too believes the shape is a blap. Assuming that the participant believes the source is likely to be correct and is therefore positively influenced by it, she will stick with her initial answer and her confidence in her judgement will increase. If the participant does not trust the source however she may be negatively influenced by its response. Negative influence would result in her lowering her confidence or changing her answer. Now let us imagine the source disagrees – it does not believe the shape is a blap. If the participant is positively influenced by the source she will become less confident in her judgement and may change her answer to match the source’s. However if she thinks the source is wrong and is negatively influenced by its response she may become more confident in her initial judgement upon seeing that the source disagrees with her.

Thus, COM is calculated for each participant and trial using the following equations:

$$COM_{keep answer}=(\alpha*Final Confidence)-(\alpha*Initial Confidence)$$

For agree trials α = 1; for disagree trials α = –1;

$$COM_{Change answer}=(\alpha*Final Confidence)+(\alpha*Initial Confidence)$$

For agree trials α = –1; for disagree trials α = 1;

We calculated average COM scores for each participant. Where there was missing data due to the source never being chosen we imputed zeros, as COM was unaffected by these sources. In Experiment 1, we find that politically similar sources and sources that were accurate on the blap task have a greater effect on COM. Participants changed their minds most when receiving information from the similar-accurate source (*M* = 40.64, *SD* = 29.45), followed by the politically dissimilar but accurate source (*M* = 35.92, *SD* = 28.04), followed by the similar-random source (*M* = 34.91, *SD* = 25.06) and finally by the dissimilar-random source (*M* = 16.71, *SD* = 31.49).

Entering COM into a two (similar/dissimilar) by two (accurate/random) repeated-measures ANOVA revealed a main effect of source accuracy (*F*(1,96) = 22.74, *p* < .001, η**_p_**^2^ = .19), a main effect of political similarity (*F*(1,96) = 15.13, *p* < .001, η**_p_**^2^ = .14) and an interaction effect (*F*(1,96) = 8.42, *p* = .005, η**_p_**^2^ = .08). The interaction was due to the random-similar source having greater influence than the random-dissimilar source (*t*(96) = 4.72, *p* < .001, *d* = .64) while there was no difference in influence between the two accurate sources (*t*(96) = 1.30, *p* = .20, *d* = .16).

In Experiment 2 we again find that the similar-accurate source had the greatest effect on COM (*M* = 38.29, *SD* = 26.70), followed by the politically dissimilar but accurate source (*M* = 30.27, *SD* = 27.42), followed by the similar-random source (*M* = 26.00, *SD* = 27.31) and finally by the dissimilar-random source (*M* = 25.25, *SD* = 27.23). The results of the ANOVA revealed a main effect of source accuracy (*F*(1,100) = 13.81, *p* < .001, η**_p_**^2^ = .12), a marginal effect of political similarity (*F*(1,100) = 3.25, *p* = .074, η**_p_**^2^ = .031), and no interaction effect (*F*(1,100) = 2.48, *p* = .11, η**_p_**^2^ = .02).
